# Morphologic and Osteometric Analysis of the Skull of Markhoz Goat (Iranian Angora)

**DOI:** 10.1155/2014/972682

**Published:** 2014-03-04

**Authors:** Nader Goodarzi, Toraj Shah Hoseini

**Affiliations:** Department of Basic Sciences, Faculty of Veterinary Medicine, Razi University, Sheikh Toosi Blvd., P.O. Box 6715685414, Kermanshah, Kermanshah Province, Iran

## Abstract

This study provides some comprehensive osteometric and morphologic descriptions of the skull region of the Markhoz goat. Totally, 17 osteometric parameters of eight skulls of Markhoz goat were measured and expressed as mean ± SD. A skull length of 18.67 ± 0.66, a cranial length of 11.1 ± 0.38, a facial length of 10.23 ± 0.76, a skull index of 47.77 ± 1.96, a cranial index of 54.04 ± 2.29, a facial index of 100.77 ± 6.85 and a foramen magnum index of 89.32 ± 14.1 were recorded. Morphologically frontal bone did not constitute the caudal extent of frontal surface; rather it was formed by the parietal bone. There were two supraorbital foramina in both sides. The prominent facial tuberosity lies dorsally to the 3rd cheek tooth. The infraorbital foramen was single on either side which was located directly dorsally to the junction of the first and second upper premolar. The orbits were round and complete and located on a frontolateral oblique plane. The basilar part of the occipital bone was surrounded by two pairs of muscular tubercles with similar size. The temporal line was continuous of the temporal crest and ran over the parietal bone. In conclusion, the morphologic and osteometric data of Markhoz goat are comparable to other ruminants.

## 1. Introduction

Markhoz goat breed, known as Angora goat in other places, was previously scattered in the Iranian provinces of Western Azerbaijan, Kurdistan, and Kermanshah [1] but is currently distributed only in a small part of Kurdistan and a few villages of Western Azerbaijan provinces. The potentiality for commercial mohair and meat production and leather industry makes Markhoz goat a popular candidate for Iranian agriculture [2]. Morphologic and morphometric studies of skull not only reflect contributions of genetic and environmental components to individual development and describe genetic and ecophenotypic variation but also are foundations of the clinical and surgical practices [3]. Furthermore, craniofacial anatomy is vital for understanding the spatial relationships of organs in this region. An important aspect of the craniofacial anatomy of any animal is the skull typology of the species [4]. Although there are such studies for the West African Dwarf goat [5] and the Kagani goat [6], there is no published information on the osteometric and morphologic feature of the skull in this species. Therefore, this study was carried out to provide some comprehensive osteometric and morphologic descriptions of the skull region of the Dwarf Markhoz goat.

## 2. Materials/Methods 

**Figure 1:**
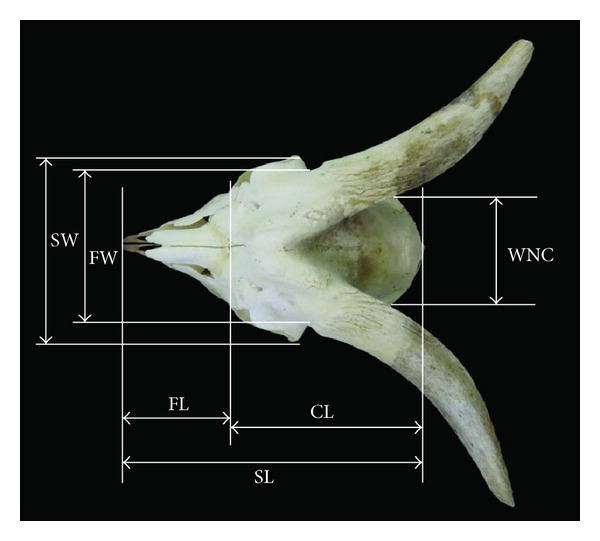
Dorsal view of the skull of Markhoz goat showing SL: skull length, FL: facial length, CL: cranial length, SW: skull width, FW: facial width, and WNC: width of neurocranium.

**Figure 2:**
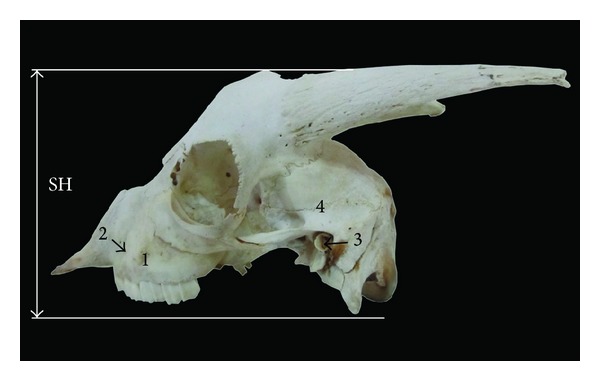
Lateral view of the skull of Markhoz goat showing SH: skull height. 1: facial tuberosity, 2: infraorbital foramen, 3: external auditory meatus, and 4: temporal fossa.

**Figure 3:**
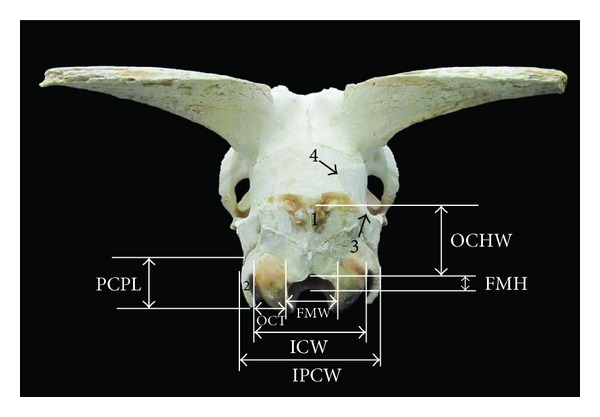
Caudal view of the skull of Markhoz goat showing FMH: foramen magnum height, FMW: foramen magnum width, ICW: intercondylar width, IPCW: interparacondylar width, OCT: occipital condyle thickness, and PCPL: paracondylar process length. 1: external occipital protuberance, 2: paracondylar process, 3: temporal crest, and 4: temporal line.

A total of eight skulls of adult Markhoz goat without any skeleton disorder were obtained from the local abattoir. The goat body weights and sexes were not considered. The heads were processed in the dissection room of Razi University using the boiling maceration techniques for skeletal preparation that has been reported [7]. Then skulls were used for morphological and osteometrical analysis. As a whole, eighteen parameters were recorded with the help of measuring scale, thread, and digital Vernier callipers using the methods described by Sarma [6] and the results were presented as means ± SD in Table 1. The parameters measured of the skulls of the Markhoz goat are described below and depicted in Figures 1–3.

### 2.1. Skull Parameters

- *Skull Length (SL). *Maximum length of the skull from the rostral tip of the incisive bones to the external occipital protuberance.
- *Skull Height (SH)*. From the level of the highest point of the frontal bone to the base of the jugular process.
- *Skull Width (SW). *Maximum breadth between two zygomatic arches.
- *Skull Index (SI)*. Skull width × 100/skull length.

### 2.2. Cranial Parameters

- *Cranial Length (CL)*. Distance from nuchal crest to the junction of the left and right nasofrontal sutures on the median plane.
- *Maximum Width of Neurocranium (MWNC)*. Distance from the most lateral point of the cranial cavity on the left to the most lateral point of the cranial cavity on the right.
- *Cranial Index (CI)*. MWNC × 100/CL.

### 2.3. Facial Parameters

- *Facial Length (FL)*. Distance from the junction of the left and right nasofrontal sutures in the median plane to the rostral end of the interincisive fissure.
- *Facial Width (FW)*. Distance between the caudal extent of the orbital rims.
- *Facial Index (FI).* FW × 100/FL.

### 2.4. Foramen Magnum Parameters

- *Foramen Magnum Height (FMH)*. Distance between the midpoints of the dorsal and ventral rims of the foramen magnum.
- *Foramen Magnum Width (FMW)*. Maximum width between two occipital condyles.
- *Foramen Magnum Index (FMI)*. Foramen magnum height × 100/foramen magnum width.
- *Occipital Triangle Height without FM (OCHW)*. Distance from the caudoventral projection of the nuchal crest to the upper rim of the foramen magnum.
- *Occipital Condyle Thickness (OCT)*. Maximum width of single occipital condyle from the most lateral extent to the foramen magnum.
- *Intercondylar Width (ICW)*. Width between the lateral borders of the occipital condyles.
- *Interparacondylar Width (IPCW)*. The greatest width between the ventromedial end of the paracondylar processes.
- *Paracondylar Process Length (PCPL)*. Length from the tip of the paracondylar process to its junction with the squamous part of the occipital bone.

## 3. Results and Discussion

### 3.1. Osteometrical Analysis

The skull index in Markhoz goat was recorded to be 47.77 ± 1.96 while it was reported to be 41.95 in Kagani goat [6] and 53.57 cm in Mehraban sheep [8] (Table 1).

The mean length and width of the skull in Markhoz goat were found to be 18.67 ± 0.66 cm and 8.91 ± 0.18 cm, respectively. On the other hand, facial length and cranial length were 10.23 ± 0.76 and 11.1 ± 0.38 cm, respectively (Table 1, Figure 1). Unlike previous studies in Mehraban sheep [8] and Kagani goat [6], the cranial length was longer than the facial portion of the skull in Markhoz goat.

Facial width and maximum width of neurocranium were 10.26 ± 0.21 and 5.99 ± 0.13 cm, respectively (Table 1, Figure 1). The facial and cranial width in Kagani goat [6] were found to be 18.28 and 4.30 cm, respectively. The result of the present study indicated that Markhoz goat has narrower facial portion and wider cranial portion in comparison with Kagani goat, while the width of skull and its cranial and facial part in studied animals were less than values reported in Mehraban sheep [8]. Furthermore, the value of height of skull in this breed was 9.63 ± 0.47 cm (Table 1, Figure 2).

In this study, the mean value of the foramen magnum index was 89.32 ± 14.1 as a result of foramen magnum width being higher than height (Table 1), while it was reported to be 103.58 in camel [9] and 102.5 in West African Dwarf goat [5].

The mean value of foramen magnum height and width in West African Dwarf goat were 1.72 and 1.67 cm, respectively [5]. These values for Kagani goat were 3.08 and 3.12 cm, respectively [6]. Therefore, according to our finding foramen magnum in Markhoz goat was smaller than Kagani goat and bigger than West African Dwarf goat (Table 1, Figure 3).

The width between lateral borders of the occipital condyles (ICW) was 4.6 ± 0.32 cm in Markhoz goat, while it was reported to be 4.09 cm and 4.0 cm in West African Dwarf goat [5] and Red Sokoto goat [4], respectively. Furthermore, width between lateral borders of paracondylar process (IPCW) and length of paracondylar process were 6.44 ± 0.23 cm and 2.09 ± 0.16 cm, respectively (Table 1, Figure 3), while these values were recorded to be 4.4 and 4.2 cm for Red Sokoto goat [4].

### 3.2. Morphological Analysis

In morphological study of the skull of Markhoz goat, macroanatomical characteristics on the frontal, lateral, nuchal, and ventral surface of the skull were considered. The following descriptions therefore cover the skull of the Markhoz goat.

#### 3.2.1. Frontal Surface

**Figure 4:**
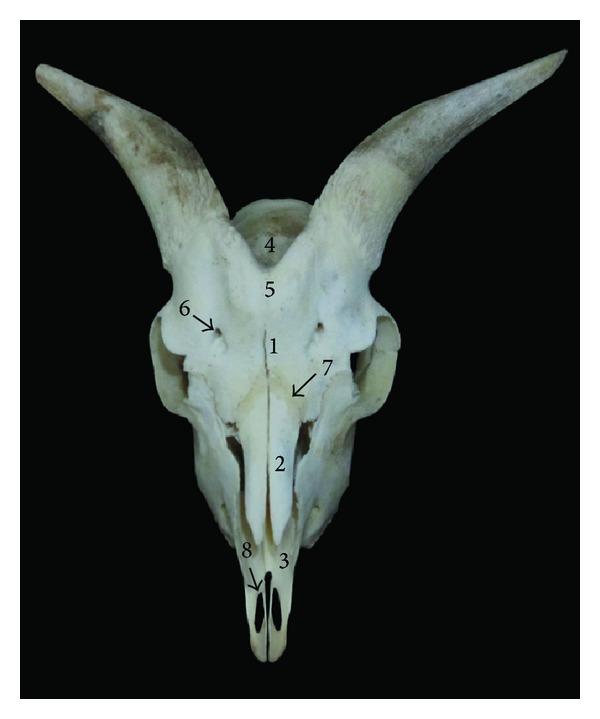
Frontal view of the skull of Markhoz goat showing 1: frontal bone, 2: nasal bone, 3: incisive bone, 4: parietal bone, 5: intercorneal prominence, 6: supraorbital foramen, 7: frontonasal suture, and 8: palatine fissure.

This surface was composed of frontal, nasal, and incisive bone. As in Kagani goat [6] the frontal bone did not constitute the caudal extent of frontal surface in Markhoz goat, whereas in cattle [10], goat [11], and mithun [12] the frontal bone extended up to the caudal extent of the skull. A large intercorneal prominence was present on the midline of the frontal bone. The frontonasal suture was “V” shaped as it was reported in Kagani goat [6] (Figure 4). The nasal bones were convex at their external surface which terminated in a sharp rostral process. The rostral end of the incisive bone was approximately blunt with two long and narrow palatine fissures. The number and location of the supraorbital foramina are dependent on the species and possibly breeds [8]. In Kagani goat [6] presence of the single supraorbital foramen was reported, while in the skull of local goat of Assam presence of two supraorbital foramina was recorded [11]. Similarly in Markhoz goat two supraorbital foramina were present on both sides. These foramina continued by the shallow supraorbital groove rostrally and caudally and were located equidistantly from the interfrontal suture, approximately 1.9 ± 0.1 cm and 4.72 ± 0.52 cm away from caudodorsal rim of the orbit and base of the cornual process, respectively.

#### 3.2.2. Lateral Surface

The prominent facial tuberosity was placed dorsal to the 3rd cheek tooth similar to ox [10], and mithun [12]. But it was placed at the junction of the 4th and 5th cheek teeth in Kagani goat [6] and dorsally to the 4th cheek tooth in Assam goat [11]. The infraorbital foramen was single on either side which was located directly dorsally to the junction of the first and second upper premolar in Markhoz goat breed (Figure 2). Cranial to this foramen a deep fossa was found lodging 1–4 foramina, whereas 4 foramina were reported in Mehraban sheep [8] and Kagani goat [6]. The temporal crest began below the cornual process and ended into a sharp and small tubercle caudolateral to the external auditory meatus, while it ended into a blunt tubercle in Kagani goat [6] and cattle [13]. The temporal fossa in Markhoz goat was deep and extensive as in goat [6] and Mehraban sheep [8]. The orbits were round and complete and located on a frontolateral oblique plane. Laterally, rostrodorsal rim of orbits had a deep notch. A deep fossa for the lacrimal sac was located on the orbital surface of lacrimal bone. The oval foramen was positioned in the caudal part of the pterygoid bone.

#### 3.2.3. Basal Surface

**Figure 5:**
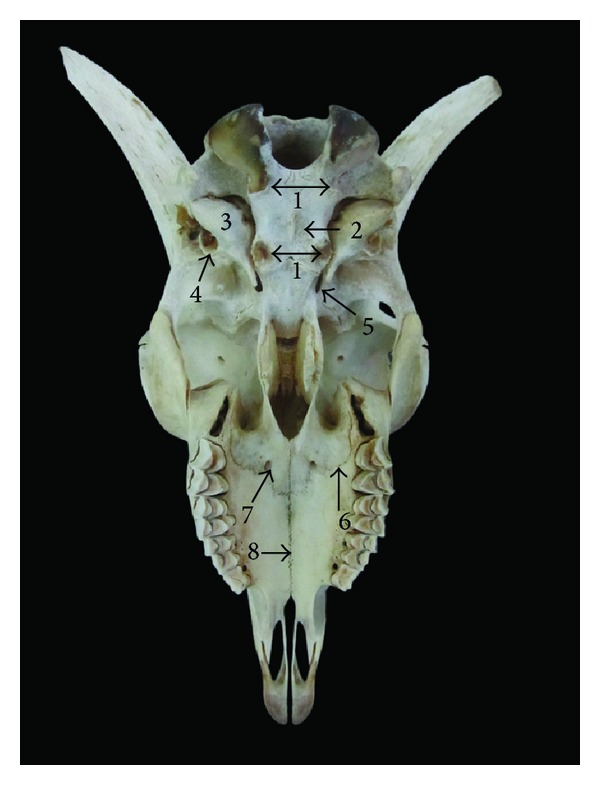
Ventral view of the skull of Markhoz goat showing 1: muscular tubercle, 2: sharp ridge on the basisphenoid, 3: tympanic bullae, 4: styloid process, 5: oval foramen, 6: transverse palatine suture, 7: greater palatine foramen, and 8: median palatine suture.

The basisphenoid bone had a sharp median ridge on its body. The basilar part of the occipital bone was surrounded by two pairs of muscular tubercles with similar size (Figure 5). It was reported that the rostral pair was larger in Mehraban sheep [8] while the caudal pair was larger in Kagani goat [6]. The tympanic bullae on the temporal bone were caudolaterally compressed and small in Kagani goat [6]. However, they were caudomedially compressed and well developed in Mehraban sheep [8]. In Markhoz goat the tympanic bulla was bilaterally compressed and well developed; also a well developed styloid process was seen. Rostral portion of the maxilla bone has become narrow and has given the palatine portion a “V” appearance and, also, transverse palatine suture was “V” shaped and serrated, lying over the greater palatine foramina. The minor palatine foramina were found to be absent. The median palatine suture is mildly serrated in rostral maxillary part (Figure 5).

#### 3.2.4. Nuchal Surface

The external occipital protuberance on the external lamina of the squamous occipital bone was prominent and sharp (Figure 3) while it was reported to be wide and blunt in Mehraban sheep [8] and sharp and pointed in Kagani goat [6]. Laterally, paracondylar process projects downward on either side and at the same level of the occipital condyle. The temporal line was continuous of the temporal crest and ran over the parietal bone.

In conclusion, the morphologic and osteometric data of Markhoz goat are comparable to other ruminants. This work has provided basic information on the skull osteometry that would be helpful for comparison with other breeds of goats and description of adaptational physiology of the Markhoz goat breed.

## Figures and Tables

**Table 1:** The various osteometrical measurements of the skull of the Markhoz goat.

Parameters	Mean ± SD
Skull parameters	
SL	18.67 ± 0.66
SW	8.91 ± 0.18
SH	9.63 ± 0.47
SI	47.77 ± 1.96
Cranial parameters	
CL	11.1 ± 0.38
MWNC	5.99 ± 0.13
CI	54.04 ± 2.29
Facial parameters	
FL	10.23 ± 0.76
FW	10.26 ± 0.21
FI	100.77 ± 6.85
Foramen magnum parameters	
FMH	1.79 ± 0.16
FMW	2.02 ± 0.19
FMI	89.32 ± 14.1
ICW	4.6 ± 0.32
IPCW	6.44 ± 0.23
OCHW	2.71 ± 0.36
PCPL	2.09 ± 0.16
OCT	1.51 ± 0.1
